# CVT in the Peripartum Period: A Diagnostic Dilemma

**DOI:** 10.1155/2020/7656232

**Published:** 2020-01-22

**Authors:** Leah Allbright, Francine McLeod, Donald Mauney

**Affiliations:** ^1^Obstetrics and Gynecology, Inova Fairfax Medical Campus, USA; ^2^Virginia Commonwealth University School of Medicine, Department of Obstetrics and Gynecology, Inova Fairfax Medical Campus, USA; ^3^Virginia Commonwealth University School of Medicine, Department of Anesthesiology, Inova Fairfax Medical Campus, USA

## Abstract

Women in the peripartum period can develop headache with a variety of etiologies that require a multidisciplinary approach if unresponsive to treatment (Stella et al. 2007). Neuroimaging needs to be undertaken even occasionally in the absence of focal neurologic signs to rule out life-threatening causes of headache. We present the case of a 23-year-old woman who presented postpartum with severe frontal headache without other neurologic symptoms. Treatment was initiated for tension type, then subsequently postdural puncture headache (PDPH), and finally preeclampsia. When CT venogram was obtained ten days later, the diagnosis of cerebral venous thrombosis (CVT) was made. She was started on anticoagulation and achieved complete recovery.

## 1. Background

It has been well established that there is a strong link between headaches and hormones, namely estrogen, which influences the severity of migraines during the menstrual cycle, pregnancy, and menopause. Additionally, the physiologic changes that occur during pregnancy put women at a higher risk for vascular-related abnormalities, such as cerebral venous thrombosis, hemorrhagic stroke, and hypertensive disorders of pregnancy like preeclampsia, posterior reversible encephalopathy syndrome, and reversible cerebral vasoconstriction syndrome [1]. The majority of headaches in pregnancy and postpartum period are tension-type and related to vasoconstriction or muscle spasm [2]. However, these serious secondary causes of headache should also be considered as they are often misdiagnosed as migraines [2, 3]. Additionally, epidural analgesia is a common form of pain relief during labor but has been associated with postdural puncture headache. This may be another contributing factor for severe headache in the postpartum period [4]. If left untreated, postdural headache can cause intracranial hypotension leading to cerebral venous thrombosis in rare cases [5]. Furthermore, the risk of CVT is also thought to be increased in those patients with sinusitis, thrombophilias, dehydration, head trauma, and pregnancy [6]. Pregnancy and postpartum status is conceivably one of the most common risk factors and misdiagnosis of CVT as an eclamptic seizure can be frequent [7, 8].

CVT can be difficult to diagnose in the absence of neuroimaging; however, neuroimaging is traditionally only obtained in the presence of focal neurologic deficits [9]. This case is presented as an example of a patient with multiple risk factors for CVT who was diagnosed with several other alternative causes of headache prior to receiving a final diagnosis of CVT. It illustrates the necessity of a multidisciplinary team approach for the diagnosis and treatment of CVT and exemplifies the need for neuroimaging for persistent headache even in the absence of focal neurologic symptoms.

## 2. Case Presentation

We present a challenging diagnostic case of a 23-year-old gravida three, para two, postpartum from a vaginal delivery. She had epidural analgesia for pain relief during her labor. Epidural administration was noted to be difficult with four attempts.

Immediately post epidural, she complained of severe, continuous, frontal headache and neck stiffness, which was initially thought to be due to muscle spasm. Her headache was persistent despite conservative measures and medications. She received an anesthesiology consultation on postpartum day one (PPD1), and although her headache lacked the traditional characteristics of postdural puncture headache (PDPH) including positional components of pain, she received a spinal patch. Her headache was unchanged immediately following the procedure. She received a neurology consultation the same day with leading differentials including tension-type headache and PDPH. Her headache then decreased in intensity several hours after spinal patch, and she requested discharge with continued conservative measures.

She was readmitted two days later with hypertensive emergency, severe frontal headache, and lab abnormalities including elevated lactate dehydrogenase, uric acid, and proteinuria. She was diagnosed with postpartum preeclampsia and treated with 24 hours of magnesium sulfate administration. Neurology consultation again was obtained, as she demonstrated no improvement in her headache with Fioricet, Toradol, Compazine, Neurontin, caffeine, or methylprednisolone. Neurology evaluation revealed completely intact neurologic exam. Funduscopic exam was not performed. Computed topography imaging without contrast on her second admission revealed possible sphenoid sinusitis, slight prominence of the pituitary gland, intraventricular air thought to be due to recent epidural injection, and slight tentorial dural calcification. The patient's headache seemingly ameliorated after treatment of her acute hypertension, and she was discharged home after much improvement in her headache.

**Figure 1:**
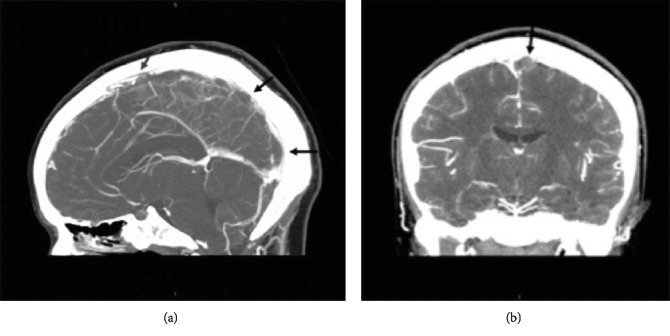
(a) CT venogram with sagittal images with filling defects of the superior sagittal sinus (arrows). (b) Coronal images with filling defects of the superior sagittal sinus (arrow).

She returned postpartum day ten (PPD10) with worsening frontal headache, neck pain, and low-grade fever. Although a neurological examination remained unchanged, consultation with internal medicine and neurology requested additional imaging. She received a CT venogram that confirmed a filling defect in the superior sagittal sinus extending into the torcula and proximal left transverse sinus, consistent with cerebral venous thrombosis (Figure 1). Her Glasgow Coma Scale (GCS) score remained 15/15. Laboratory tests including platelet count, protein C, protein S, antithrombin III, autoantibodies, and antiphospholipid antibodies were unremarkable. Lupus anticoagulant was indeterminate. The only abnormal finding was heterozygosity of methylenetetrahydrofolate reductase.

## 3. Treatment

The patient was immediately treated with therapeutic heparin and then transitioned to warfarin with an INR goal of 2-3. A treatment course of 6 months of anticoagulation was planned with repeat imaging to assess for resolution. Her pain was controlled with IV hydrocodone, methocarbamol, Gabapentin, and Fioricet.

## 4. Outcome and Follow-Up

**Figure 2:**
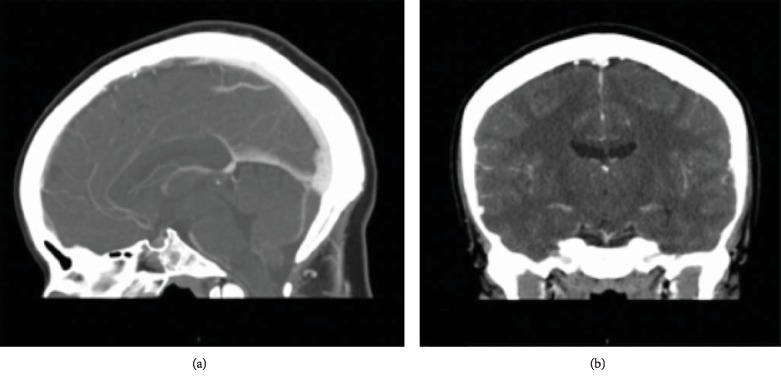
(a) CT venogram with sagittal images resolution of filling defects. (b) Coronal images with resolution of filling defects.

The patient was discharged home hospital day five, PPD15. Her headache had resolved by the day of discharge. Follow-up after one year and four months revealed continued therapeutic anticoagulation without neurologic symptoms. Hematology referral was given but not pursued by the patient. Five months later, the patient had repeat CT venogram which demonstrated resolution of cerebral venous thrombosis (Figure 2).

## 5. Discussion

Headache in the peripartum and postpartum period can have varied etiologies with occasional severe life-threatening causes including CVT. Thrombosis and thromboembolism is a leading cause of pregnancy-associated deaths, and CVT has a reported incidence of 11.6 per 100,000 deliveries per year, accounting for 6-64% of all pregnancy-related strokes [10]. Risk factors for the development of CVT include the hypercoagulable state of pregnancy, infection including sinusitis, head trauma, hypertensive conditions, and most recently, dural punctures after spinal analgesia [5, 6, 11]. Loss of CSF after dural puncture decreases intracranial pressure, leading to a compensatory cerebral venous and arterial dilation (Monro-Kellie hypothesis), reducing the venous blood flow in dural sinuses by 50% following lumbar punctures [12]. The pull on the cerebral vessels caused by a pressure gradient causes damage to the vessel endothelium and the clotting factors in cerebrospinal fluid increase, completing Virchow's triad to lead to thrombosis [13]. Bijral et al. detailed a case report of a postpartum patient complicated by CVT after PDPH who additionally made a complete recovery following anticoagulation, [5]; however; unlike our patient, she had typical postdural headache symptoms and focal neurological deficits prompted immediate imaging.

Our patient initially presented with severe, acute onset headache immediately after epidural placement. The patient lacked traditional symptoms of PDPH including positional components; however, its temporality with epidural analgesia made PDPH the most likely initial etiology. Dural puncture is cited to be a rare complication of 1.5% of all epidural anesthesia catheter placements, and in approximately 50% of these patients, they develop headache [14]. A more recent study cited a possible incidence of dural puncture as high as 76-85% of epidural placements [15]. Despite conservative medical measures and a spinal patch procedure, the patient did not initially improve. Due to the persistent headache, neurology and anesthesiology consultation was obtained, and additional imaging was recommended despite normal neurological exam. It should be noted that on multiple occasions, the patient did not perceive her symptoms to be troublesome enough to warrant continued hospitalization and she refused additional imaging. In her second admission for severe preeclampsia, she received a CT without contrast that demonstrated a sphenoid sinusitis without evidence of midline shift, or hemorrhage. The American Heart Association and American Stroke Association Scientific Statement on the diagnosis and management of CVT recommends imaging of the cerebral venous system in those with suspected CVT, preferably head CT without contrast, for evaluation of patients with new headache, focal neurological abnormalities, seizures, or mental status changes. However, head CT can be normal in 20% of cases of CVT, and magnetic resonance imaging or angiography has been found to be more sensitive to the diagnosis of CVT [6, 16]. Therefore, MRI, despite patient reluctance, should have been sought earlier in the clinical course as it is the most sensitive study for detection of CVT [17].

Our patient also had a history of postpartum preeclampsia in a prior pregnancy and presented postpartum with a severe headache, new-onset hypertension, and an abnormal antenatal protein-to-creatinine ratio. Although unclear whether she had worsening proteinuria, she met the criteria for diagnosis of preeclampsia traditionally defined as new-onset hypertension with new-onset proteinuria, or lab abnormalities that constitute HELLP syndrome in the absence of proteinuria [18]. Preeclampsia has been considered an additional risk to the development of CVT because of its associated potential disruption of the blood–brain barrier, vasogenic edema, and focal areas of impaired venous drainage with subsequent thrombus formation [18–21].

Finally, the patient was at risk for development of CVT by presenting in the immediate postpartum period. A case-control study conducted by Silvis et al. utilizing cases of CVT across 5 academic hospitals and controls from the Dutch MEGA study detected 41 cases of CVT diagnosed during pregnancy or postpartum (up to 12 weeks) and found that, when adjusted for pregnancy and the postpartum period, the highest attributable risk of development of CVT was during the first 6 weeks postpartum (adjusted odds ratio: 18.7; 95% CI: 8.3-41.9) and, surprisingly, little association between pregnancy itself and CVT (adjusted odds ratio: 1.2; 95% CI: 0.6-2.3) [8]. Therefore, CVT should be strongly considered in the postpartum period for those with intractable headache, as this may confer a tenfold increased risk of CVT than nonpregnant or postpartum women of similar age.

The patient therefore demonstrated multiple risk factors for CVT making the diagnostic evaluation for CVT challenging. While headache is the most frequent presenting symptom, the characteristics are not uniform. CVT headache is generally subacute in onset but may have a variable location including unilateral, frontal, occipital, or temporal laterality [22]. Focal neurological deficits usually are present in 44% of cases, of which motor weakness is the most common [3, 19]. The diagnosis of CVT in our patient was elusive. Not only did she have waxing and waning headache but she also failed to develop seizures or focal neurologic findings. Funduscopic exam was excluded. If funduscopic exam had been performed and papilledema present, then intracranial hypertension syndrome may have been a leading diagnosis. Additionally, without initial imaging, it is difficult to delineate a specific timeline of events. Did CVT precede her presenting headache or did her combination of risk factors and events predispose to thrombosis that manifested as CVT? A pooled, systematic review of CVT suggested that clinical presentation at the time of treatment is strongly associated with long-term clinical outcomes. For example, patients with headache alone are 3.9 times more likely to achieve long-term excellent outcomes, which can be problematic because many patients are only diagnosed because of a decreasing neurological exam [23].

Our patient's thrombophilia screening immediately postpartum excluded possible hematologic predisposition to clotting [11], although she did not present for hematologic testing prior to or after pregnancy. While she was heterozygous for the MTHFR gene and hyperhomocysteinemia promotes vascular damage of the endothelium and has been linked to venous thrombosis, there is no established link with heterozygosity for the MTHFR gene and thrombosis. The A1298C allele may not possess disease risk unless homozygous or present with other alleles [21]. In fact, the most recent European Stroke Organization guideline for the diagnosis and treatment of cerebral venous thrombosis recommends against routine thrombophilia testing as all studies are observational with high risk of bias and inconsistent conferred risk of reoccurrence [24].

Our patient was anticoagulated for a period of six months postpartum to prevent thrombus growth and promote recanalization, in conjunction with the recommended treatment course of CVT associated with pregnancy and may be pursued even in the presence of intracranial bleeding [19, 20, 25, 26]. Our patient demonstrated normal venous architecture in follow-up scans with resolution of her thrombosis. The European Stroke Organization recommends that patients with a previous CVT should avoid estrogen-containing compounds for family planning. Furthermore, if pregnancy is desired, prophylactic low-molecular-weight heparin should be utilized during the antenatal and immediate postpartum period [24].

In summary, cerebral venous thrombosis is a rare but life-threatening condition that presents a diagnostic challenge in the peripartum period. A multidisciplinary team approach with the departments of obstetrics, anesthesiology, internal medicine, and neurology is necessary to optimize the evaluation and management toward a favorable outcome. Neuroimaging with CT angiography or MRI should be obtained in patients with persistent headache even in the absence of focal neurologic findings.
